# Development and validation of the impact of dry eye on everyday life (IDEEL) questionnaire, a patient-reported outcomes (PRO) measure for the assessment of the burden of dry eye on patients

**DOI:** 10.1186/1477-7525-9-111

**Published:** 2011-12-08

**Authors:** Linda Abetz, Krithika Rajagopalan, Polyxane Mertzanis, Carolyn Begley, Rod Barnes, Robin Chalmers

**Affiliations:** 1 Mapi Values, Adelphi Mill, Grimshaw Lane, Bollington, Cheshire, SK10 5JB, UK; 2 Sunovion Pharmaceuticals Inc. 84 Waterford Drive Marlborough, MA 01752, USA; 3 Independent consultant, Shrewsbury, MA, USA; 4 Indiana University, School of Optometry, 800 East Atwater Avenue, Bloomington, IN 47405, USA; 5 Health Care Value Evidence, 6817 Lahontan Drive, Fort Worth, TX 76132, USA; 6 Clinical Trial Consultant, 2097 East Lake Road, Atlanta, GA 30307, USA

**Keywords:** Dry eye, Sjögren's Syndrome, non-Sjögren's keratoconjunctivitis sicca, health-related quality of life, treatment satisfaction, symptoms, impact, patient-reported outcome, questionnaire

## Abstract

**Objective:**

To develop and validate a comprehensive patient-reported outcomes instrument focusing on the impact of dry eye on everyday life (IDEEL).

**Methods:**

Development and validation of the IDEEL occurred in four phases: 1) focus groups with 45 dry eye patients to develop a draft instrument, 2) item generation, 3) pilot study to assess content validity in 16 patients and 4) psychometric validation in 210 subjects: 130 with non-Sjögren's keratoconjunctivitis sicca, 32 with Sjögren's syndrome and 48 controls, and subsequent item reduction.

**Results:**

Focus groups identified symptoms and the associated bother, the impact of dry eye on daily life and the patients' satisfaction with their treatment as the central concepts in patients' experience of dry eye. Qualitative analysis indicated that saturation was achieved for these concepts and yielded an initial 112-item draft instrument. Patients understood the questionnaire and found the items to be relevant indicating content validity. Patient input, item descriptive statistics and factor analysis identified 55 items that could be deleted. The final 57-item IDEEL assesses dry eye impact constituting 3 modules: dry eye symptom-bother, dry eye impact on daily life comprising impact on daily activities, emotional impact, impact on work, and dry eye treatment satisfaction comprising satisfaction with treatment effectiveness and treatment-related bother/inconvenience. The psychometric analysis results indicated that the IDEEL met the criteria for item discriminant validity, internal consistency reliability, test-retest reliability and floor/ceiling effects. As expected, the correlations between IDEEL and the Dry Eye Questionnaire (a habitual symptom questionnaire) were higher than between IDEEL and Short-Form-36 and EuroQoL-5D, indicating concurrent validity.

**Conclusion:**

The IDEEL is a reliable, valid and comprehensive questionnaire relevant to issues that are specific to dry eye patients, and meets current FDA patient-reported outcomes guidelines. The use of this questionnaire will provide assessment of the impact of dry eye on patient dry eye-related quality of life, impact of treatment on patient outcomes in clinical trials, and may aid in treatment effectiveness evaluation.

## Introduction

The Dry Eye Workshop defined dry eye as a "multifactorial disease of the tears and ocular surface that results in symptoms of discomfort, visual disturbance, and tear film instability with potential damage to the ocular surface" [1,2]. It is accompanied by detrimental effects on patients' health-related quality of life (HRQL) [3-6] and vision-related quality of life [7]. Dry eye or non-Sjögren's keratoconjunctivitis sicca (non-SS KCS) is a condition due to lacrimal and/or meibomian gland dysfunction leading to diminished production or increased evaporation of tears [1,8]. It may be associated with Sjögren's Syndrome (SS), which is a systemic autoimmune disorder in which chronic inflammation of the lacrimal and salivary glands, that eventually leads to insufficient tear production, and the characteristic clinical features of dry eyes and dry mouth [1,9,10]. There are two basic forms of SS [1,11]: primary SS, the disease by itself that is not associated with any other illness, and secondary SS, that develops in the presence of another autoimmune disease such as rheumatoid arthritis, lupus or psoriasis [1,11,12]. In susceptible individuals, exacerbating factors such as systemic medications that decrease tear production or environmental conditions that increase tear evaporation may lead to an increase in the severity of symptoms. Thus, dry eye is a chronic condition that is heterogeneous not only in aetiology, but also in severity.

Multiple studies have shown that clinical tests can be poorly associated with the changes in symptoms as the disease progresses and the self-perceived severity of the condition [13-18], although recent studies have shown higher correlations with tear osmolarity [18] Other conditions such as allergy, basement membrane disease or conjuntivochalasis could present as dry eye, with symptoms of ocular irritation. Thus, a validated questionnaire that fully assesses symptoms together with the effect of dry eye on daily life is indicated [4,19,20].

Because dry eye symptoms can occur without clinical signs of tissue damage, it has been considered a symptom-based condition, especially in mild to moderate cases[1,21]. It is often under-diagnosed relative to the patients' assessment [17], particularly among the elderly and women. An incidence study in the elderly showed a 21.6% increase in dry eye over a ten-year period, which increased with age and was greater in women [22]. However, patients with clear signs of dry eye may report few symptoms, perhaps due to sensory damage of the ocular surface. Thus, refining patient reported outcomes to track the natural history of the condition, its variability and its effect on daily life becomes critical [18].

Some of the common treatment methods for SS include artificial tears, anti-inflammatory drugs, and balanced diet and exercise to overcome pain and fatigue [23,24]. Treatment of non-SS KCS is generally confined to tear film replenishment and stabilization through use of artificial tears, gels and ointments, ergonomic modifications and punctual occlusion [23]. In moderate to severe non-SS KCS, patients report treatments to be of limited value [13], and often become frustrated with their treatment course, are forced to repeatedly visit doctors and specialists, and ultimately seek alternative treatments [25].

Multiple dry eye-specific questionnaires exist that aim to assess frequency and severity of the dry eye symptoms or to help in dry eye diagnosis and screening [13-16,26-30]. While the Ocular Surface Disease Index (OSDI) addresses the impact of dry eye on vision-related functioning and dry eye symptoms in terms of severity, it covers only some of these aspects and is therefore unlikely to describe the full impact and burden of dry eye and its treatment on patients' everyday life [6]. Only the IDEEL covers all relevant domains of dry eye including dry eye symptoms and dry eye-related quality of life domains of the patients' life that might be impacted (i.e. visual functioning activities, psychological, social and cognitive aspects), as well as treatment satisfaction [4,19,20,31,32].

The purpose of this study was to develop and validate a comprehensive patient-reported outcomes (PRO) instrument, the Impact of Dry Eye on Everyday Life (IDEEL) that extensively evaluates dry eye symptoms and all the aspects of patients' daily life impacted by the condition and its treatment.

## Methods

### Dry eye expert clinicians

Dry eye expert clinicians on the team provided expertise and took part extensively in the decisions throughout the process of development and finalisation of the questionnaire. They gave their approval at each of the milestones of the development and finalisation process. The questionnaire was also reviewed and agreed by the clinical investigators.

### Development of the questionnaire

#### Phase 1: Patient focus groups

Six focus groups were conducted in the United States (Alabama, California, Indiana and Minnesota) and Canada (Toronto and Waterloo) with non-SS KCS (four groups) and SS (two groups) subjects. Patients were recruited in hospital-based clinics and private practices. Clinical investigators at each study site recruited subjects using the International Classification of Diseases, Ninth revision, Clinical Modification (ICD-9CM) for the non-SS KCS subjects and the San Diego criteria for SS subjects (which includes a positive salivary gland biopsy) [33]. To be eligible, subjects had to be aged over 18 years and had to have dry eye symptoms in the previous four weeks; they were excluded if they had a punctual occlusion within the past 60 days or if they had experienced a change in systemic medication regimen within the last 30 days. Subjects signed consent forms prior to study participation and were compensated for their time. The focus groups were performed by trained moderators using an interview guide specifically designed for the purpose to help moderators lead the discussions, and to ensure conformity across focus groups. Focus groups were recorded and subsequently transcribed verbatim.

Qualitative analysis of transcripts was performed using methods derived from Grounded Theory, with patients' quotes coded and organized into themes or concepts [34]. Analysis was performed using Atlas.ti software version 6.2 [35]. To verify adequate sample size and the full coverage of the research topic, saturation was studied. Saturation is defined as the point at which no new concepts or information emerge with the addition of more patient data [36-38]. Saturation was determined by following concepts and information that arose per focus groups ranked in the chronological order they were conducted.

#### Phase 2: Concept elicitation and item generation

Using grounded theory methods, concepts and sub-concepts were elicited from the analysis of transcripts. All concepts and sub-concepts mentioned by subjects and related to symptoms, daily impact, or treatment satisfaction bother/convenience were included in a comprehensive set of items that were generated to develop the initial version of the questionnaire.

#### Phase 3: Pilot study/Cognitive debriefing

The feasibility of the pilot version of the questionnaire was subsequently tested in dry eye subjects in the US and Canada in an individual interview setting. Half of the subjects in this phase had been involved in the focus groups and the remaining half was recruited using the same method as in Phase 1. Subjects completed the pilot version and were questioned regarding their general impressions of the questionnaire; its comprehensiveness; the clarity of the instructions, items and response choices; and their interpretations and opinions of the relevance of each question. Subjects were also asked to provide suggestions on how to reword the instructions, questions and response options.

### Finalisation, scoring and psychometric validation of the questionnaire

#### Study population and design

To participate in the psychometric validation study [13], outpatients subjects had to be at least 18 years old, must have had an eye exam in the past 18 months, and a confirmed diagnosis of either non-SS KCS or SS (except for the controls). Five optometrists and one ophthalmologist participated as clinical investigators at six study sites. ICD-9CM codes and the San Diego criteria (which includes a positive salivary gland biopsy) were used to identify non-SS KCS and SS subjects, respectively [33]. Potential study subjects were screened by the investigators by telephone, with a series of questions that ensured the presence of dry eye symptoms in the previous 4 weeks; patients were excluded if they wore contact lenses, had undergone refractive surgery, had a punctual occlusion within the past 60 days, or had experienced a change in systemic medication regimen within the past 30 days. Control subjects were recruited from lists of patients who did not have ICD-9CM diagnostic codes for dry eye. During the telephone screening, these subjects had to have responded negatively to the question, "Do you think you have dry eye?" and that they have "never" or "rarely" had dry eye symptoms or used artificial tears. Finally, at least two thirds of the control subjects recruited had to be older than 35 years to ensure the control population characteristics were as close as possible as the ones of the patients. Subjects also had to be literate in English, willing and able to complete a series of questionnaires twice over a two-week period and willing to undergo clinical testing for dry eye as part of the study. Consent forms were signed by all the subjects prior to study participation and they were all compensated for their time.

Eligible patients underwent two visits: a baseline visit and a second visit 2 weeks later. Informed consent was obtained from subjects at the baseline visit [13].

### Patient-reported outcome questionnaires and clinical tests

The subjects completed the following questionnaires at baseline and two weeks later, in the following order: the pilot version of the IDEEL questionnaire, the Medical Outcomes Study Short-Form-36 Health Survey (SF-36) a 36-item general measure of health status [39], the revised Dry Eye Questionnaire (DEQ 2001) [27,39,40] and the EuroQoL (EQ-5D), a 5-item general utility measure of health attributes [41]. The DEQ 2001 is a revision of the earlier DEQ questionnaire, which was validated in a large unselected clinical population and against dry eye diagnosis [27,40]. Subjects also completed a demographic form at the first visit and the Dry Eye Change Scale, a 3-item change questionnaire that assesses change in overall dry eye symptom status, at the second visit.

To try to assess whether there was a relationship between these clinical measures and the PROs measures, the following clinical tests were performed by the investigators during the first visit: Snellen visual acuity, Schirmer 1 tear test, fluorescein tear break-up time, corneal fluorescein staining, and conjunctival lissamine green staining.

### Psychometric Analysis

#### Construct validity

The percentages of subjects from the total study population who chose response options "not applicable" or "none of the time" to the IDEEL items at baseline were reviewed: items with percentages greater than 70% were considered for deletion, as were items with high levels of missing data (> 20%).

Principal components analysis (PCA) with promax rotation analysis aided in the development of item-dimension structures and item reduction of the pilot version of the IDEEL [42]. Factors were retained when eigenvalue was greater than 1 [42]. A threshold for factor loading of 0.40 was fixed for the PCA: items that did not load well (≤ 0.40) with their own factor and items that loaded > 0.40 on more than one factor were considered for deletion [43]. To be included in the factor analysis, subjects had to have completed all items in each scale. If patients in the focus groups and content validity indicated an item was important and expert clinicians also endorsed the item as relevant to dry eye, the item was retained regardless of statistical results.

Multi-trait Analysis was also performed to determine the correlation between each item and the dimension to which it belonged [43]. The analysis was performed twice: before and after item reduction. As with the PCA step, an item was considered for deletion if it did not correlate with its own dimension at ≥ 0.40 (item convergent validity) or if it correlated higher with a dimension other than its own (item discriminant validity) [43]. Floor and ceiling effects were also investigated for each of the items.

The following properties were assessed on the final version of the IDEEL, after item reduction, finalisation and scoring.

#### Reliability

Internal consistency reliability and reproducibility ('test-retest reliability') were examined. A Cronbach's alpha coefficient of ≥ 0.70 was considered acceptable for internal consistency [44]. Test-retest reliability was evaluated by examining the Interclass Correlation Coefficients (ICCs) between visit one (week 0) and visit two (week 2) for patients who reported stability in their dry eye symptoms in the previous 2 weeks. An ICC of ≥ 0.70 was considered acceptable for test-retest reliability [45].

#### Concurrent validity

Correlations (Pearson coefficients) between the general health measures (SF-36, EQ-5D) and the specific questionnaire DEQ were studied and compared to similar dimensions (i.e. covering a same concept) in the IDEEL. Concurrent validity was supported if similar dimensions or items in the SF-36, EQ-5D and DEQ were substantially correlated (r ≥ 0.40) with the IDEEL [46].

#### Known group validity

Known group validity was assessed by examining differences in IDEEL baseline scores for groups of patients with different levels of dry eye severity for each severity assessment method. Three types of severity assessments were made during the study: diagnosis severity of the patients recruited (i.e. control, non-SS KCS or SS), clinician report of severity and patient self-report of severity.

#### Clinical validity

The correlation between the IDEEL and clinical tests previously listed was examined to assess clinical validity.

Statistical analyses were conducted using Statistical Analysis Software version 8.2 and Multi-trait Analysis Program - Revised software version 1.0 [43]. Analyses were conducted using parametric tests. For all tests, a significance level of 0.05 (two-sided) was used unless otherwise indicated. When reducing the number of items in the questionnaire, both statistical results and the clinical relevance of items were considered prior to deletion.

### Conceptual framework

Following PCA and Multi-trait analysis findings, the final conceptual framework of the questionnaire was developed with input from clinical experts, and based on the importance of the concepts and sub-concepts from patients' perspective.

## Results

### Development of the questionnaire

The focus groups had 6 to 10 participants in each; the total population consisted of 45 patients: 30 with non-SS KCS and 15 with SS. Age ranged from 20 to 79 years (mean age = 58 ± 14 years). The majority of the patients were female (91%), Caucasian (85%), with at least a high school diploma or General Educational Development (GED) diploma (86%). Time since dry eye diagnosis ranged from 5 months to 25 years. Ninety percent of the patients self-rated the severity of their dry eye as mild/moderate (58%) or severe (32%). High blood pressure and arthritis were the health conditions other than dry eye most frequently experienced by subjects (22% and 20%, respectively).

The following concepts and sub-concepts emerged from the focus group analysis as relevant to patients experience with dry eye: vision-related symptoms and their bothersomeness, including burning/heat sensations, dryness/irritation, moisture-related symptoms, pain-related symptoms, tired eyes, eye appearance, swelling, tearing, light/wind sensitivity; daily life impact, including physical, daily activities, work, relationships, cognitive, emotions, leisure and social impact, visual-aid impact, tiredness/sleep, appearance/aesthetics and general impacts; and treatment experiences and satisfaction in terms of inconvenience, effectiveness and frequency.

Saturation was achieved for dry eye symptoms and these symptom sub-concepts, as well as for all of the overarching concepts of dry eye daily life impact and for the aforementioned sub-concepts.

Concepts and sub-concepts were selected that were clinically important (as discussed with the dry eye expert clinicians) and also important from a patient's perspective (as emerged from the focus group discussions). An initial 116-item questionnaire was developed that was organized around the following modules: dry eye symptom-bother (37 items); dry eye impact on daily life, including impact on daily activities (21 items), emotional impact due to dry eye (32 items), impact on work due to dry eye (8 items); satisfaction with overall treatment (9 items) and satisfaction with eye drops (9 items). The items were generated in US English.

The questionnaire was subsequently comprehension tested in 16 subjects (mean age: 63 years; range: 41-79 years). The subjects were mainly female (81%), Caucasian (87%) and had at least a high school diploma or equivalent (94%). The 116-item questionnaire was completed in 18.5 minutes on average (range: 11-35 minutes). Overall, subjects expressed positive comments regarding the questionnaire. Based on their reported levels of understanding and their suggestions, minor wording changes were made to the instructions and questions. In total, two questions were removed, four sets of questions were combined and three questions were split into six questions, yielding 112 questions and six hypothesised dimensions as described above. This questionnaire was then fielded in the psychometric validation study.

### Psychometric validation

#### Study population demographics

The demographics of the validation study subjects are presented in Table 1. The population included 210 adult subjects: 130 with non-SS KCS, 32 with SS, and 48 controls. The majority of the population was Caucasian female and the mean age was 51 years (range: 20-89 years). Follow-up pairwise t-tests showed that the group of controls was significantly younger (39 years) than both the non-SS KCS (55 years) and SS groups (58 years); the latter two groups did not differ statistically by age.

**Table 1:** Demographic characteristics of the psychometric validation study population (n = 210)

Characteristics	Control (n = 48)	Non-SS KCS* (n = 130)	SS* (n = 32)
**Sex (n (%))**			

Male	13 (27)	27 (21)	3 (9)
Female	35 (73)	103 (79)	29 (91)

**Age (Year)**			

Mean	39.2	55.2	58.3
Standard deviation	11.8	15.3	11.8
Range	20.0-66.0	22.0-89.0	34.0-80.0

**Ethnicity (n (%))**			

Caucasian	34 (71)	106 (82)	31 (97)
African-American	6 (13)	12 (9)	1 (3)
Hispanic/Spanish-American	5 (10)	5 (4)	0 (0)
Asian/Oriental/Pacific Islands	1 (2)	6 (5)	0 (0)
Other	2 (4)	1 (1)	0 (0)

**Highest level of education (n (%))**			

High school diploma or less	9 (19)	23 (18)	6 (19)
Some college	13 (27)	47 (36)	8 (25)
College degree	15 (31)	29 (22)	11 (34)
Graduate/postgraduate	8 (17)	31 (24)	5 (16)
Other	3 (6)	0 (0)	2 (6)

* Non-SS KCS, Non Sjögren keratoconjunctivitis sicca; SS, Sjögren Syndrome [13]

### Item Reduction, descriptive statistics and construct validity

Given that dry eye symptom-bother, dry eye impact on daily life and dry eye treatment satisfaction are conceptually distinct, it was hypothesised that each module could be processed as single distinct sub-questionnaire [47-49]. PCA was thus conducted on each of these modules separately. The impact on work due to dry eye dimension was both included and excluded from factor analyses due to smaller sample sizes for the work-related items. The analyses mentioned below were conducted on the total study population (n = 210).

For the Dry Eye Impact on Daily Life module, the eigenvalues obtained with the PCA for the Impact on Daily Activities and Emotional Impact due to Dry Eye dimensions confirmed two distinct dimensions, with eigenvalues > 1. For the Impact on Work Scale, the eigenvalues suggested one dimension. Four items did not load on any of the factors, and were retained as individual items for conceptual reasons (i.e. items of importance for the patients and/or expert clinicians; these items were about wearing contact lenses, wearing make-up, flying on an airplane and feeling helpless about dry eye). The Dry Eye Symptom-Bother module yielded a single factor, indicating a single dimension. The eigenvalues for the Treatment Satisfaction module suggested two dimensions: Satisfaction with Treatment Effectiveness and Treatment-Related Bother/Inconvenience. Two items did not load on any of the factors, and were retained as individual items for conceptual reasons (items about treatment frequency).

Only the Dry Eye Treatment Satisfaction module showed a floor effect, which was low (5.84% for the total dry eye sample). Ceiling effects were present for all IDEEL modules with the exception of the Dry Eye Symptom-Bother module (0.6%). Acceptable ceiling effects were evident for the remaining dimensions (14.9% for Impact on Daily Activities, 13.7% for Emotional Impact due to Dry Eye for those who were working, 13.4% for Impact on Work due to Dry Eye and for those on treatment, 1.6% for Treatment Satisfaction and 15.2% for Treatment-related Convenience/Bother.

Based on the PCA results, the item descriptive statistics, construct validity and face validity results, a total of 55 items were removed from the pilot version of the questionnaire: 27 items were removed because more than 70% of the patients answered "None of the time", "Not at all" or "Not applicable" to items; 1 item was removed because 77% of the patients did not answer the item; 25 items were removed according to the loading factor analysis results; and 4 items were combined into 2 because they were highly correlated (> 0.90) and had a similar wording ("burning" with "stinging"; "ache" with "sore").

**Figure 1:**
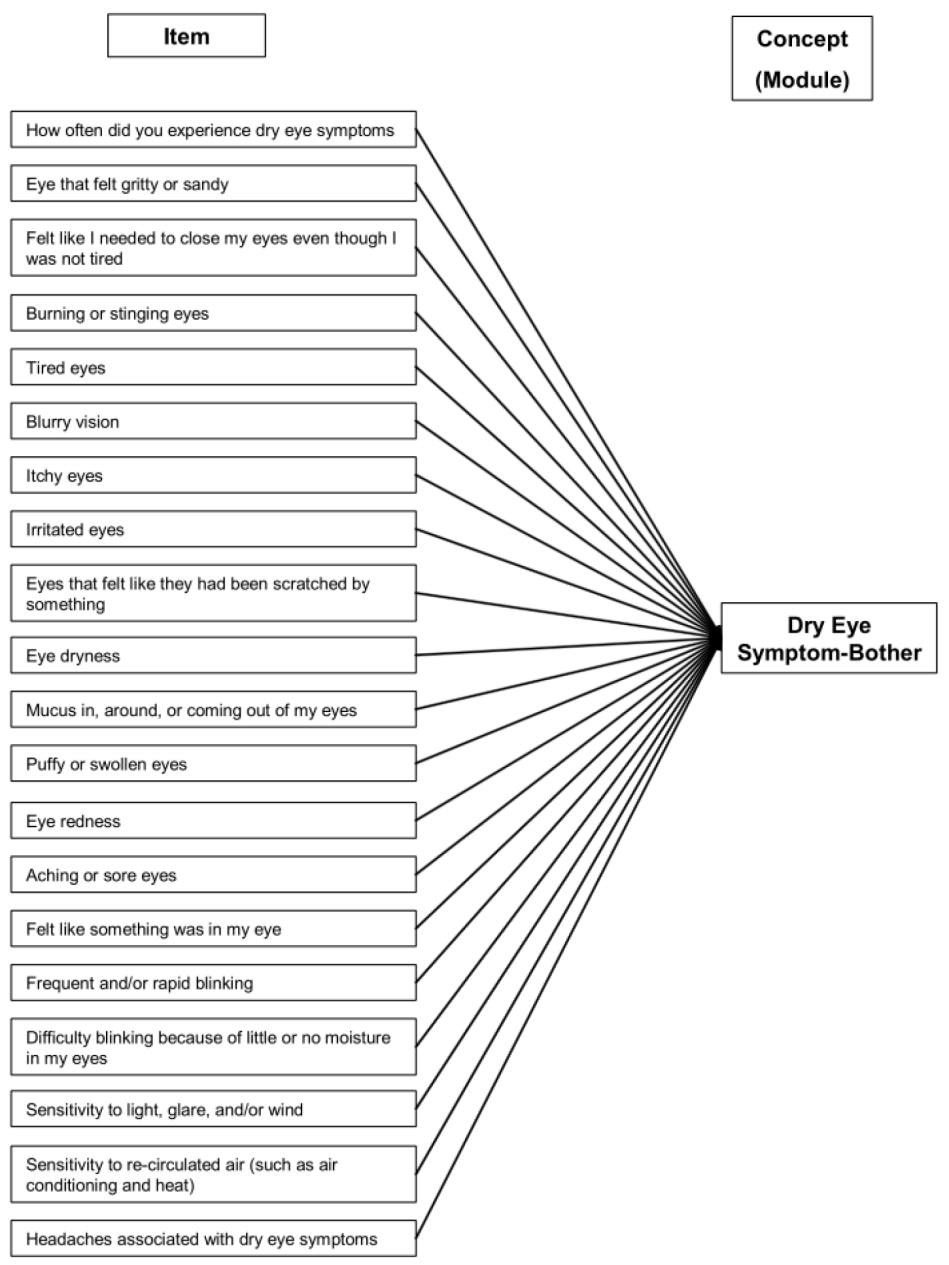
**Conceptual framework of the final version of IDEEL: Dry Eye Symptom-Bother module**.

**Figure 2:**
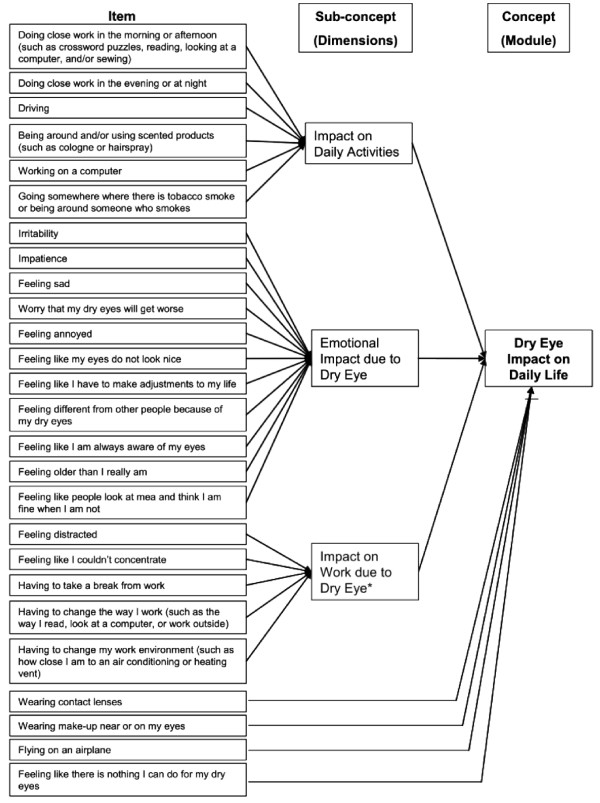
**Conceptual framework of the final version of IDEEL: Dry Eye Impact on Daily Life module**. * One additional item about working status introduces the items of the dimension. The item is not considered during the scoring of the IDEEL.

**Figure 3:**
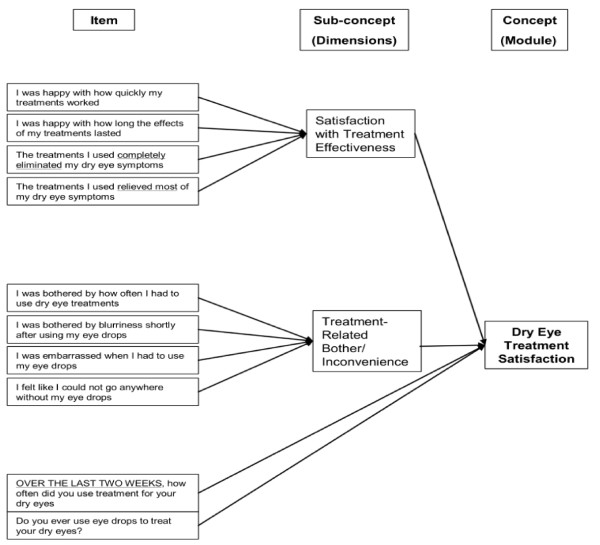
**Conceptual framework of the final version of IDEEL: Dry Eye Treatment satisfaction module**.

Thus, the final IDEEL consists of 57 items organised into 3 different modules. The Dry Eye Symptom-Bother module constituted a single dimension (20 items). A 4-point Likert scale was used for all items except "frequency of experience of dry eye symptoms" that was scored on a 5-point Likert scale. An option indicating the symptom was not present was also available. The Dry Eye Impact on Daily Life module was composed of 3 dimensions covering Impact on Daily Activities (9 items), Emotional Impact due to Dry Eye (12 items) and Impact on Work due to Dry Eye (6 items); 4 individual items and a working status item were part of the module. A 5-point Likert scale was used for all the items except "work status" that was scored on a dichotomous "Yes" or "No" scale. The Dry Eye Treatment Satisfaction module was composed of two dimensions covering Satisfaction with Treatment Effectiveness (6 items) and Treatment-Related Bother/Inconvenience (4 items); the 2 individuals items were part of the module. A 5-point Likert scale was used for all the items except "eye drop use", that was scored on a dichotomous "Yes" or "No" scale. The questionnaire was named IDEEL: Impact of Dry Eye on Everyday Life. The conceptual framework of each of the three modules of the questionnaire is described in Figure 1, Figure 2 and Figure 3.

Scores for each dimensions ranged from 0 to 100. Higher scores for the dimensions of the Dry Eye Impact on Daily Life module indicated less impact on daily activities, work and emotions; higher scores for the Symptom-Bother dimension indicated greater bother due to symptoms; higher scores for Satisfaction with Treatment Effectiveness dimension indicated greater satisfaction with treatment effectiveness; higher scores with Treatment-related Bother/Inconvenience indicated less treatment-related bother or inconvenience.

### Item convergent validity/discriminant validity

All items of the "Emotional Impact due to Dry Eye", "Symptom-Bother" and "Satisfaction with Treatment Effectiveness" met the discriminant validity criteria (each item should correlate higher with its own dimension than with the other dimensions).

### Reliability

The internal consistency and test-retest reliability results for the IDEEL are presented in Table 2. Internal consistency reliability was good (Impact on Daily Activities, Impact on Work; Satisfaction with Treatment Effectiveness) to excellent (Dry Eye Symptom-Bother; Emotional Impact). Although the Treatment-related Bother/Inconvenience Scale only just surpassed the reliability criterion with a Cronbach's alpha of 0.70, this indicates strong internal reliability as the scale consists of 4 fairly heterogeneous items. All the dimensions of the IDEEL showed good test-retest reliability, ICC ranging from 0.70 to 0.88.

**Table 2:** Internal consistency (Cronbach's alpha) and test-retest reliability (Intraclass Correlation Coefficients, ICC) properties of the IDEEL

Dimensions of the IDEEL	Cronbach's α coefficient (N*)	ICC (N**)
Dry Eye Symptom-Bother module*		

Dry eye Symptom Bother	0.97 (209)	0.88 (167)

Dry Eye Impact module*		

Impact on Daily Activities	0.86 (209)	0.77 (167)

Emotional Impact due to Dry Eye	0.94 (209)	0.83 (167)

Impact on Work Scale	0.86 (128)	0.70 (106)

Dry Eye Treatment Satisfaction module*		

Satisfaction with Treatment Effectiveness	0.87 (125)	0.70 (111)

Treatment-related Bother/Inconvenience	0.70 (139)	0.80 (130)

* Number of patients who completed more than half of the items in the respective dimensions

** Number of patients for whom a score could be calculated and who were stable between the 2 visits according to the Dry Eye Change Scale (N = 167)

### Concurrent validity

Correlations between the IDEEL and SF-36 and EQ-5D were low across the majority of dimensions (ranges: 0.04-0.45 and 0.09-0.36 respectively), as expected (Table 3). In contrast, the dimensions of the IDEEL were more highly correlated with the items of the DEQ (range: -0.05-0.83), indicating the concurrent validity of the IDEEL (Table 3). The strength of the associations between the IDEEL dimensions and DEQ items was as expected. In general, the IDEEL dimensions were most highly correlated with items pertaining to eye dryness and eye discomfort. The Dry Eye Symptom-Bother module of the IDEEL was the dimensions most highly correlated with all of the DEQ items (0.21-0.83). All the IDEEL correlations showed the lowest correlations with the item "DEQ Watery eyes: Frequency in the past week" (-0.05-0.21).

**Table 3:** Concurrent validity of the IDEEL with the SF-36, EQ-5D and Dry Eye Questionnaire (Pearson coefficient correlations)

Questionnaires	Modules of the IDEEL					
	
	Dry Eye Symptom-Bother*	Dry Eye Impact on Daily Life			Dry Eye Treatment Satisfaction	
		
		Impact on Daily Activities*	Emotional Impact due to Dry Eye*	Impact on Work due to Dry Eye*	Satisfaction with Treatment Effectiveness*	Treatment-related Bother/ Inconvenience*
**Short Form-36**						
Physical Functioning	-0.33	0.30	0.35	0.04	0.06	0.35
Role Physical	-0.35	0.30	0.36	0.24	0.15	0.37
Bodily Pain	**-0.39**	**0.34**	0.39	0.22	0.14	0.31
Vitality Index	-0.36	**0.34**	0.40	**0.31**	0.24	0.38
General Health Perceptions	-0.38	0.32	**0.45**	0.18	0.17	**0.40**
Social Functioning	-0.31	0.31	0.40	0.27	0.11	0.35
Role Emotional	-0.27	0.29	0.33	0.20	0.12	0.29
Mental Health Index	-0.32	0.33	0.40	0.13	**0.30**	0.27
Physical Component Scale	-0.37	0.31	0.38	0.16	0.09	0.37
Mental Component Scale	-0.26	0.29	0.36	0.23	0.23	0.26

**EuroQol-5D**						
EQ-5D Quality of Life Score	**-0.36**	**0.31**	**0.35**	**0.24**	**0.23**	**0.30**
EQ-5D VAS Health Thermometer	-0.34	0.30	0.35	0.22	0.09	0.22

**Dry Eye Questionnaire**						
All items (correlation min; correlation max)	0.21; 0.83	-0.19; -0.65	-0.11; -0.69	-0.07; -0.68	-0.05; -0.48	-0.06; -0.60

* Number of patients ranging from 203 to 209 for Dry Eye Symptom-Bother, Impact on Daily Activities and Emotional Impact due to Dry Eye; ranging from 130 to 131 for Impact on Work due to Dry Eye; ranging from 131 to 137 for Satisfaction with Treatment Effectiveness; ranging form 148 to 154 for Treatment -Related Bother/Inconvenience

Bold font correlations are the highest correlations between the IDEEL scales and the SF-36 or EQ-5D scales.

### Known group validity

Differences between severity groups were assessed by patient-recruited diagnosis (control, non-SS KCS and SS), clinician report of symptoms (none, mild, moderate and severe) and patients' self-rating of severity ("don't have dry eye," "very mild/mild dry eye," "moderate dry eye" and "severe/very severe dry eye"). For the analysis, the very mild and mild groups were collapsed, as were the severe and very severe groups. For the Satisfaction with Treatment Effectiveness Scale, there was no "don't have dry eye" group, as patients without dry eye symptoms did not complete this section of the questionnaire.

**Figure 4:**
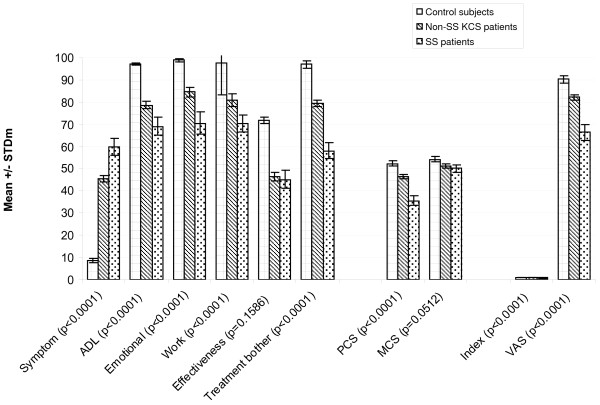
**Known group validity of the IDEEL, SF-36 and EQ-5D dimensions by patient-recruited severity of the diagnosis**. Non-SS KCS = non-Sjögren keratoconjunctivitis sicca; SS = Sjögren Syndrome; IDEEL, Impact of Dry Eye on Everyday Life; SF-36, Short-Form-36; EuroQoL-5D, EQ-5D; Symptom, Symptom-Bother dimension; ADL, Impact on Daily Activities dimension; Emotional, Emotional Impact due to Dry Eye dimension; Work, Impact on Work due to Dry Eye dimension; Effectiveness, Satisfaction with treatment effectiveness dimension; Treatment bother, Treatment-related bother/inconvenience dimension; PCS, Physical Component Scale; MCS, Mental Component Scale; Index, EQ-5D items (score ranges from 0 to 1); VAS, Visual Analogue Scale.

**Figure 5:**
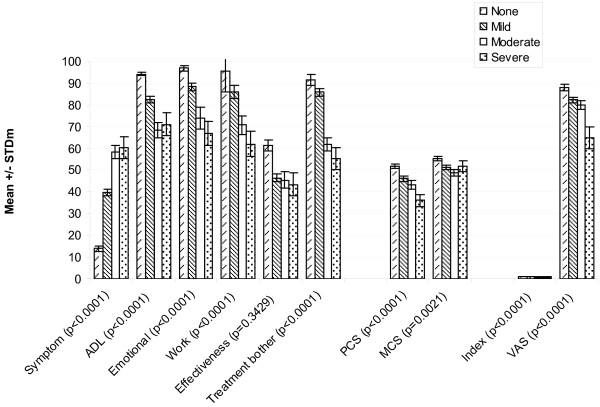
**Known group validity of the IDEEL, SF-36 and EQ-5D dimensions by clinician-rated severity**. IDEEL, Impact of Dry Eye on Everyday Life; SF-36, Short-Form-36; EuroQoL-5D, EQ-5D; Symptom, Symptom-Bother dimension; ADL, Impact on Daily Activities dimension; Emotional, Emotional Impact due to Dry Eye dimension; Work, Impact on Work due to Dry Eye dimension; Effectiveness, Satisfaction with treatment effectiveness dimension; Treatment bother, Treatment-related bother/inconvenience dimension; PCS, Physical Component Scale; MCS, Mental Component Scale; Index, EQ-5D items (score ranges from 0 to 1); VAS, Visual Analogue Scale.

**Figure 6:**
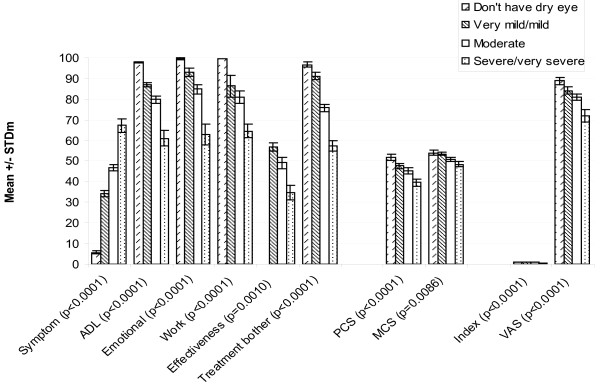
**Known group validity of the IDEEL, SF-36 and EQ-5D dimensions by patient-rated severity**. IDEEL, Impact of Dry Eye on Everyday Life; SF-36, Short-Form-36; EuroQoL-5D, EQ-5D; Symptom, Symptom-Bother dimension; ADL, Impact on Daily Activities dimension; Emotional, Emotional Impact due to Dry Eye dimension; Work, Impact on Work due to Dry Eye dimension; Effectiveness, Satisfaction with treatment effectiveness dimension; Treatment bother, Treatment-related bother/inconvenience dimension; PCS, Physical Component Scale; MCS, Mental Component Scale; Index, EQ-5D items (score ranges from 0 to 1); VAS, Visual Analogue Scale.

Significantly different (p < 0.0001) mean scores were observed between different levels of severity in all the IDEEL dimensions except Satisfaction with Treatment Effectiveness, regardless of whether the criterion used was recruited severity, clinician-rated severity, or patient-rated severity (Figure 4, Figure 5 and Figure 6, respectively). For the SF-36, significant differences between the various severity levels were noted in the Physical Component Scale scores across the 3 different severity criteria. Significant differences across severity levels for the Mental Component Scale score were observed only among the clinician-rated severity and patient-reported severity. In observing the EQ-5D results, significant differences in mean dimension scores at the varying severity levels were also consistently noted across all criterion measures. The Symptom-Bother Scale performed the best of all the IDEEL scales, as expected.

### Clinical validity

Correlations between the clinical signs and the Impact on Daily Activities, Emotional Impact due to Dry Eye, Impact on Work due to Dry Eye, Treatment-related Bother/Inconvenience and Symptom-related Bother dimensions of the IDEEL were low and statistically significant (p < 0.05) for most of them except between Snellen visual acuity, bulbar redness and fluorescein break-up time and the Work dimension (Table 4). The Symptom-related Bother and Treatment-related Bother/Inconvenience dimensions had the highest correlation with clinical signs overall (up to 0.37) while the Satisfaction with Treatment Effectiveness scale did not correlate significantly with any of the clinical signs (Table 4).

**Table 4:** Clinical validity of the IDEEL (Pearson correlation coefficients)

Clinical tests	IDEEL Modules					
	
	Dry Eye Symptom-Bother*	Impact Daily Life			Dry Eye Treatment Satisfaction	
		
		Impact on Daily Activities*	Emotional Impact due to Dry Eye*	Impact on Work due to Dry Eye*	Satisfaction with Treatment-Effectiveness*	Treatment-related Bother/ Inconvenience*
Snellen visual acuity	0.22**	-0.25**	-0.24**	-0.09	-0.07	-0.25**
Bulbar redness	0.18**	-0.15**	-0.22**	-0.18	0.01	-0.25**
Schirmer 1 tear test (mm)	-0.30**	0.14**	0.24**	0.11	0.09	0.37**
Fluorescein break up time (sec)	-0.22**	0.17**	0.20**	0.22**	0.01	0.28**
Overall corneal fluorescein staining rating	0.31**	-0.21**	-0.25**	-0.30**	0.06	-0.32**
Conjunctival lissamine green staining rating	0.30**	-0.17**	-0.25**	-0.29**	0.03	-0.31**

* Number of patients ranging from 206 to 209 for Dry Eye Symptom-Bother, Impact on Daily Activities and Emotional Impact due to Dry Eye; ranging from 129 to 131 for Impact on Work due to Dry Eye; ranging from 135 to 137 for Satisfaction with Treatment Effectiveness; ranging form 151 to 154 for Treatment -Related Bother/Inconvenience

** p < 0.05

## Discussion

Many studies have shown that dry eye signs poorly correlate well with symptoms, although late day symptoms show moderate correlations with some signs [13] and composite scores including both signs and symptoms may improve diagnosis of the condition [18]. This suggests a need for more emphasis on PRO in dry eye in order to fully assess the condition and effect of treatments [3,50]. Existing PRO instruments focus on the ability to identify and diagnose dry eye, assess the prevalence of the condition, and assess the severity and frequency of symptoms. With novel treatments under development, the need evolved for an instrument to assess the impact of change in the dry eye condition. From a clinician's perspective, this will help in setting realistic expectations and in measuring the degree of improvement in individual patients and the remaining issues. From a regulatory perspective, such an instrument will allow a standardised method to assess treatment effectiveness in primarily symptom-based conditions such as dry eye so that claims can be supported with evidence in the form of PRO.

Despite being developed prior to the FDA PRO guidance, the IDEEL instrument was developed in accordance with standards outlined [51], which requires demonstration of content validity and saturation. The IDEEL was developed based on patient input and is the first dry eye PRO questionnaire to formally demonstrate saturation of relevant concepts, therefore supporting its content validity. The SS patients were quicker to express the detrimental impacts dry eye had on every aspect of their lives when compared to non-SS KCS patients during the focus group discussions, however saturation was achieved on the overall population. To further support content validity, when decisions were made to delete items, psychometrics and the clinicians' and patients' own opinions were considered. Multi-Trait and PCA analyses confirmed the hypothesised structure of the IDEEL as 3 distinct modules. The final IDEEL comprises 57 items organised into 3 modules, Dry Eye Symptom-Bother, Dry Eye Impact on Daily Life, and Dry Eye Treatment Satisfaction, that allow a comprehensive evaluation of the burden of the dry eye condition on patients. The assumption about the pre-defined structure of the IDEEL as 3 distinct modules and the lack of data from PCA and Multi-trait analyses on the questionnaire as a whole could be raised as limitations, as the purpose of these analyses is to allow the definition of the structure and organisation of the instrument. However, satisfaction, which is defined as an emotive evaluation which enables the assessment of the appropriateness of the perceived quality of treatment with expectations [52], is conceptually different from HRQL, that covers physical, psychological, social functioning, and somatic sensations [47]. Similarly, it is now acknowledged that symptoms are a PRO distinct from HRQL [49]. In light of these definitions, our assumption appears to be legitimate.

Psychometrically, the IDEEL final dimensions are reliable and valid. In particular, when comparing results to the SF-36 and EQ-5D, the IDEEL consistently outperformed these generic measures in distinguishing between patients' reported severity, clinician-rated severity and patient-rated severity groups, as one would expect with a condition-specific scale (data not shown) [20]. The generic measures appear capable of distinguishing between more severe categories of patients (i.e., moderate versus severe), but not capable of distinguishing between mild and moderate levels. In contrast, the IDEEL dimensions are able to distinguish between the majority of severity levels, with the exception of the Treatment-related Satisfaction module; however treatment satisfaction might not be expected to differ by severity of condition alone. One might be surprised with the low correlation level between some of the IDEEL dimensions and the SF-36 (such as "impact on work" of IDEEL and "role physical" of SF-36; "emotional impact" of IDEEL and the mental component (or some of the related scales) SF-36). The SF-36 role-physical items focus on impact due to their 'physical health. We believe that when people respond, they interpret 'physical health' to mean a disease or bodily impairments (e.g. torso, limbs) and probably do not think of dry eye as a 'disease' nor think of the eye as being 'bodily'. Further cognitive debriefing of SF-36 would be required to confirmed or not this hypothesis.

In our testing across known diagnostic groups, results were fairly consistent across methods of defining severity (self-assessment, clinician assessment, or recruited diagnosis). Given the relative lack of clarity in the field on how to define dry eye severity precisely, we used these methods to define severity. We would recommend using this type of approach in any tests of known groups or clinical validity, since the use of insensitive or non-specific clinical criteria that do not relate to symptoms could result in concluding that a questionnaire is not discriminative or responsive, when in reality it is the clinical criterion used to define severity that is not discriminative or responsive. This issue is particularly important in dry eye because no single clinical measure is widely accepted as the 'gold standard' in predicting dry eye symptoms and the impact of dry eye on patients' quality of life, [53] although recent evidence by Sullivan, Lemp and coworkers suggests that tear film hyperosmolarity may correlate better than other clinical tests for the condition [18,54]. Because clinical judgments remain central in the assessment of dry eye severity, the same team has proposed the use of composite severity index associating osmolarity testing with traditional tests of the traditional clinical tests in order to classify dry eye severity as accurately as possible [18]. The likelihood that dry eye symptoms stem from a number of aetiologies also confounds the effort to start the diagnostic process from the signs rather than the symptoms. Determining that the patient is experiencing sufficient symptoms to warrant further diagnosis and treatment is a more effective clinical starting point for such a complex condition. Hence the importance of using a PRO instrument such as the IDEEL in conjunction with the clinical tests to properly assess the improvement of patients' dry eye condition.

Further development work is ongoing to allow the use of the IDEEL as an estimate of the overall health impact of dry eye and improvement by treatment. A recent study established the minimal clinically important difference of the IDEEL dry eye Symptoms-Bother dimension in mild, moderate and severe dry eye [32]. A 12-point shift in the IDEEL Dry Eye Symptom-Bother dimension score was determined to be the clinical important difference relating to a global change in dry eye condition after implementing tear replacement drops [32]. Research in assessing the overall dry eye health burden on the patients is ongoing, including its comparison to other diseases, and highlights the impact of dry eye condition in patients in terms of utilities outcomes [55].

The focus groups in the development and in the validation of the IDEEL included US and Canadian patients. At the same time in Europe, a PRO instrument, the Ocular Surface Disease (OSD), was being developed based on French dry eye patients' outcomes. Similar concepts and sub-concepts were identified from this study, supporting the validity of the concepts that had emerged from the focus groups, as well as the appropriateness of the items to European patients [56,57]. It is likely that in other parts of the world, no different issues associated with dry eye will emerge, but this should be verified before being implemented in a study. The use of the IDEEL in Europe is made possible given the availability of linguistically validated translations in the different European languages. So far, currently validated or in development languages include German, Spain, French for France, English for UK, Polish, Italian Portuguese (Brazil), Simplified Chinese (Singapore), and Spanish for US.

The use of the IDEEL in other different studies will continue to provide further data to obtain further information on the importance of specific items for specific dry eye patients with different aetiologies. It may also allow the further reduction of items, while ensuring all the important concepts to patients are evaluated, in case of developing a briefer instrument for use in clinical practice. The items of the IDEEL are likely to be appropriate for specific sub-groups of dry eye patients; due to the similarity of the symptom-bother and daily life impact reported by patients with meibomian gland dysfunction (data not shown) [58]. However, to ensure all issues of relevance in this condition are covered, future studies and saturation assessment would be required.

In conclusion, the IDEEL is a reliable and valid questionnaire relevant to the issues that are specific to dry eye patients and it meets the FDA PRO guidelines. The IDEEL is the only comprehensive instrument that was designed to assess the entire gamut of the impact of dry eye on patient outcomes: domains of patients' everyday life, treatment satisfaction, and amount of bother by symptoms. IDEEL will aid in the assessment of DE symptom severity and impact, and will complement information gathered through traditional clinical exams and measures. The continued use of the IDEEL instrument, either as a whole or as specific modules, will provide assessment of the impact of dry eye on patient dry eye-related quality of life that may aid treatment selection and dry eye management in clinical practices.

## Competing interests

RC is a paid consultant or has research funding from Alcon Research Ltd and CIBA Vision that has now been absorbed into Alcon, Inspire Pharmaceuticals, Bausch & Lomb, Johnson & Johnson Vision Care. RC and CB have developed and published the Dry Eye Questionnaire. LA, KR, PM, CB, and RB have no competing interests.

## Authors' contributions

At the time of the study, KR and RB were employees at Alcon Research Ltd, Fort Worth, TX; PM was an employee at Mapi Values, Boston, MA. LA developed study protocols, analysed qualitative and quantitative data, developed IDEEL, wrote a first draft of the manuscript and reviewed subsequent manuscript versions. KR developed study protocol, developed IDEEL, and reviewed each version of the manuscript. PM conducted interviews and qualitative analysis, developed IDEEL, wrote the study report that was the basis for the manuscript, and reviewed each version of the manuscript. CB contributed clinical insights into to the study protocol, IDEEL development, and manuscript, recruited patients for both phases of the study, and reviewed each version of the manuscript. RC contributed clinical insights into the study protocol, IDEEL development and manuscript, recruited patients for both phases of the study; CB also conducted some of the qualitative interviews and attended the focus group in Birmingham, AL, and was involved in the first editing and reviewing of each version of the manuscript. RB reviewed the study protocol, questionnaires and manuscripts.

All authors read and approved the final manuscript.
